# Pneumococcal Colonization in the Familial Context and Implications for Anti-Pneumococcal Immunization in Adults: Results from the BINOCOLO Project in Sicily

**DOI:** 10.3390/ijms18010105

**Published:** 2017-01-06

**Authors:** Fabio Tramuto, Emanuele Amodio, Giuseppe Calamusa, Vincenzo Restivo, Claudio Costantino, Francesco Vitale, on behalf of the BINOCOLO Group

**Affiliations:** 1 Department of Health Promotion Sciences and Mother-Child Care “G. D’Alessandro”—Hygiene Section, University of Palermo, 90127 Palermo, Italy; amoema79@libero.it (E.A.); giuseppe.calamusa@unipa.it (G.C.); vincenzo.restivo@unipa.it (V.R.); claudio.costantino01@unipa.it (C.C.); francesco.vitale@unipa.it (F.V.); 2 Clinical Epidemiology Unit, University Hospital “Paolo Giaccone”, 90127 Palermo, Italy

**Keywords:** *Streptococcus pneumoniae*, conjugate vaccine, serotypes, schoolchildren, family, carriage, colonization, horizontal transmission, real-time PCR

## Abstract

The spread of *Streptococcus pneumoniae* within families has been scarcely investigated so far. This feasibility study aimed to estimate the prevalence of pneumococcal carriage in school-aged children and co-habiting relatives and to explore the potential link between the family environment and the sharing of pneumococcal serotypes covered by the vaccine. Oropharyngeal samples of 146 subjects belonging to 36 different family groups were molecularly tested for pneumococcal detection and serotyping. The overall prevalence of pneumococcal carriage was 65.8% (*n* = 96/146), whereas it was higher among schoolchildren (77.8%, *n* = 28/36); subjects of seven years of age had the highest odds of being colonized (odds ratio, OR = 5.176; *p* = 0.145). Pneumococcal serotypes included in the 13-valent conjugate vaccine formulation were largely detected in the study population and multiple serotypes colonization was considerable. Factors relating to a close proximity among people at the family level were statistically associated with pneumococcal carriage (OR = 2.121; *p* = 0.049), as well as active smoking habit with a clear dose-response effect (ORs = 1.017–3.326). About half of family clusters evidenced similar patterns of carried pneumococcal serotypes and the odds of sustaining a high level of intrafamilial sharing increased with household size (ORs = 1.083–5.000). This study highlighted the potential role played by the family environment in sustaining both the circulation and horizontal transmission of pneumococcus.

## 1. Introduction

*Streptococcus pneumoniae* is recognized as a commensal bacterium of the human upper respiratory tract and the acquisition of nasopharyngeal carriage is considered to be the first step toward respiratory and invasive pneumococcal disease (IPD) [1]. IPD occurs most frequently in children of five years of age and under and in the elderly [2,3], and, in general, the high rates of IPD in younger children correspond to higher prevalences of pneumococcal carriage [4,5]. The increasing frequency of bacterial colonization in children may grow up to 90% in the first years of life, whereas a proportional decrease of carriage prevalence by age has been described [6,7].

In 2004, Sicily introduced the 7-valent pneumococcal conjugate vaccine (PCV7) into the regional childhood immunization programme [8,9], reaching within a few years consistently high coverage levels, near to 95%; in 2010, the vaccination schedule switched to the 13-valent formulation (PCV13) [10] that aimed to provide a broader protection against IPD.

In accordance to other experiments conducted in Europe and USA [11,12,13,14], in Sicily the immunization programme led to a consistent reduction of IPD in vaccinated children [15]. Due to the burden of IPD in older adults [16,17] however, since 2014, the vaccination was also routinely recommended for adults aged 65 years and older and for individuals from all age-groups at particularly high risk for IPD, following a sequential schedule including a first dose of PCV13 eventually associated, one year later, with one dose of the polysaccharide 23-valent (PPV23) formulation.

However, despite the free and active offer of vaccination, coverage rates in adults are still very low, following a trend also observed in other countries [16,18,19,20].

Several authors have reported that high rates of vaccination coverage in childhood may result in the herd immunity among unvaccinated subjects, protecting against disease caused by serotypes included in vaccine formulations [21], but also preventing nasopharyngeal colonization [22].

Nevertheless, the potential effect of PCV on pneumococcal carriage is still debated and represents a critical issue in terms of the positive pressure that may lead to a vaccine-induced serotype replacement and the potential impact on the chain of transmission from vaccinated children to unprotected adults/elderly in the social context [23].

In this sense, the international literature seems to suggest that the family environment may play an active role in the circulation of current and emerging pneumococcal serotypes spreading through household contacts [24].

Surveillance studies may provide insight into the dynamics of *S. pneumoniae* carriage within families and household members. This is also potentially related to multiple pneumococcal strains, which may co-exist in the upper respiratory tract of children in the first stages of their life [25,26].

Here we present a feasibility study that aimed to evaluate the oropharyngeal pneumococcal carriage in a population of school-aged children and their co-habiting relatives, in order to identify the contributing risk factors and determinants of sharing the PCV-included pneumococcal serotypes into the family setting, as well as their implications on pneumococcal vaccination strategies dedicated to adults/elderly.

## 2. Results

### 2.1. Subjects Characteristics

The study enrolled a total of 36 different schoolchildren (out of 189 invited students; 19.0%) of which 12 attended first grade, 7 attended second grade, and 17 attended third grade of the selected primary schools. The family-based group consisted of 110 subjects (out of 146; 75.3%), all members of the 36 students’ family units (Table 1).

In general, participants to the study belonged to the lower-middle social class; for the adult relatives, only 37.0% (*n* = 34/92) declared to have a job and only 29.3% (*n* = 27/92) had completed an upper secondary education or obtained an academic degree.

More than half of the study population ≥12 years of age (52.2%; *n* = 48/92) was non-smoker; 33.3% (*n* = 16/48) reported to have been exposed to passive smoking. Active smoking habits were documented in 47.8% of adult family members (*n* = 44/92), who started smoking, on average, at 15 years of age and consuming about 15 cigarettes per day. In total, 15.7% of subjects (*n* = 23/146) were regularly involved in sport activities (Table 1).

With regard to medical history, most family members declared to suffer of a pre-existing disease (63.6%; *n* = 70/110), with hypertension as the most common among adults (32.4%; *n* = 22/68), while only 11.1% of schoolchildren (*n* = 4/36) and 5.6% of their siblings (*n* = 1/18) presented allergy-related symptoms.

### 2.2. Vaccination Status

Data on immunization coverage were available for the entire study population. Pneumococcal vaccination coverage was high among schoolchildren (86.1%; *n* = 31/36), whereas rates among households consistently differed between siblings aged <12 years (72.2%; *n* = 13/18) and parents (2.2%; *n* = 2/92).

Overall, 86.9% (*n* = 40/46) of subjects <12 years of age (including schoolchildren and siblings) completed the pneumococcal vaccination schedule (96.8%; *n* = 30/31 and 76.9%; *n* = 10/13, respectively); in more detail, 77.4% of schoolchildren (*n* = 24/31) received a complete schedule of the 7-valent vaccine, while seven (22.6%) were vaccinated with PCV7 + PCV13.

Coverage rates for hexavalent and MMR were ≥90% among children, either among students or siblings, while the percentages ranged between 80.6% and 83.3% for varicella vaccine; in general, rates reported among adults were negligible.

As expected, meningococcal C vaccination coverage levels in the cohorts of children included in our study population were low because of the vaccine introduction into the regional schedule in 2010; the level of immunization against influenza was even lower (5.5%; *n* = 8/146) and most of the vaccinated subjects were elderly (87.5%; *n* = 7/8).

### 2.3. Pneumococcal Carriage

Oropharyngeal colonization by *S. pneumoniae* was evaluated and an overall prevalence of 65.8% (*n* = 96/146) was observed (Table 2). On the whole, the odds of carrying the pneumococcus among students was about two times higher than that observed in the group of family members (OR = 2.161; *p* = 0.084), and the probability was the highest among children aged seven years (OR = 5.176, *p* = 0.145). The comparison by gender showed a slight association with females (OR = 1.143; *p* = 0.704).

Factors relating to a close proximity among people were summarized in the “crowding index” and this supported a borderline statistically significant association with pneumococcal carriage (OR = 2.121; *p* = 0.049).

Active smoking potentially correlated to pneumococcal colonization (OR = 1.900; *p* = 0.143) with a clear dose-response trend (OR range: 1.017–3.326) (Table 2). Also, pneumococcal carriage was more frequently detected (OR = 1.286, *p* = 0.591) in those not engaged in regular physical activity, as well as in individuals with pre-existing respiratory diseases (OR = 1.500, *p* = 0.251).

**Figure 1:**
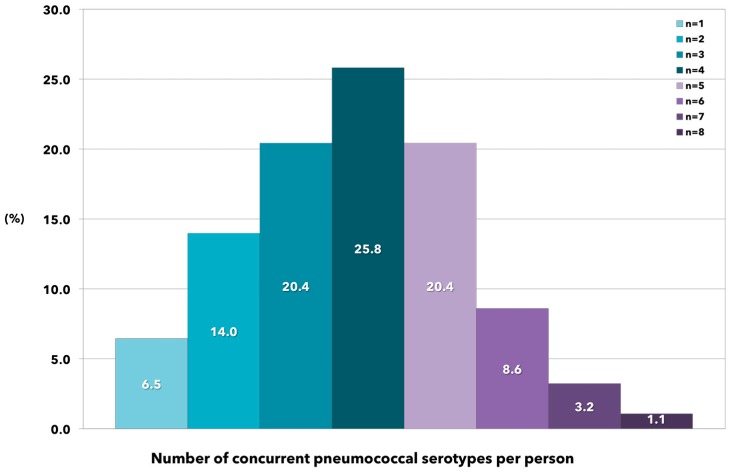
Relative proportions of concurrent PCV13 serotypes identified in oropharyngeal swab specimens collected from healthy subjects positive for *Streptococcus pneumoniae* (co-colonization).

The number of PCV13 serotypes carried per person was also investigated. Almost all the 96 colonized study participants harbored more than one serotype, and the relative proportions followed a normal distribution (Figure 1). In general, about half of all colonized individuals had three to four different serotypes in their oropharynx, whereas less than seven percent of persons carried only one pneumococcal serotype; notably, about 30.0% of them harbored five to eight different pneumococcal serotypes.

**Figure 2:**
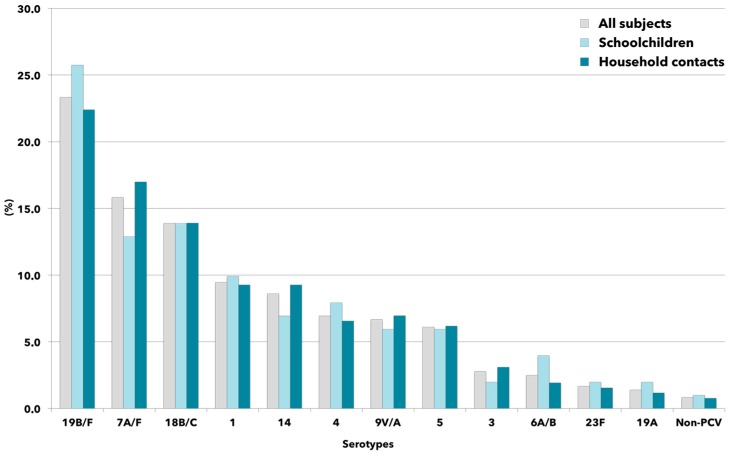
Distribution of PCV13 serotype (in ranking order) of colonizing pneumococcal strains collected from schoolchildren and household contacts. Bar graphs indicate the percentage of each serotype determined in relation to the total number of identified serotypes in the study period. Labels including the character “/” identify potential cross-reacting molecular targets.

Figure 2 illustrates the distribution of pneumococcal serotypes detected in oropharyngeal swab specimens. In general, no differences were found between schoolchildren and household contacts, with the serotypes 19 B/F, 7 V/A and 18 B/C being the most common (range: 12.9%–25.7%), whereas the frequency of detection of the other PCV13 serotypes was consistently lower than 10%.

Table 3 reports the level of intrafamilial sharing of pneumococcal serotypes within each family context, expressed as percentages.

In detail, the denominator was calculated as the total number of positive rt-PCR tests for PCV13 serotypes among all individuals in the household, including the student. Conversely, the numerator consisted of the whole number of positive rt-PCR results, among all other members of the same family, for those pneumococcal serotypes identified in oropharyngeal specimen collected from the related student.

For each family-cluster, the ratio was arbitrarily assumed to provide an estimate of measurement for the intrafamilial spread of pneumococcus.

According to the calculated percentages, the whole family setting was divided into quartiles, 16 out of 36 family groups fell between the 3rd and 4th quartile, and the score 50% was arbitrarily chosen in order to identify two different groups defined at “low” or “high” pneumococcal intrafamilial sharing.

Thus, the univariate analysis was performed with the aim of exploring the potential role of specific socio-demographic contexts suggestive of crowded environments in the households and still positively associated with pneumococcal carriage (Table 4).

In particular, the “crowding index” suggested a potential correlation with the intrafamilial sharing of pneumococcus (OR = 1.667; *p* = 0.475), and the odds increased with the increasing household size (ORs range: 1.083–5.000).

## 3. Discussion

Studies on pneumococcal colonization in children have been widely undertaken [21,27,28,29]. However, the contribution of PCV immunized children to bacterial spread among their unvaccinated co-habiting relatives has not been investigated so far.

This study evaluated the pneumococcal colonization among school-aged children and their households and it aimed to define both the prevalence of carriage and the potential spread of bacterial serotypes within family clusters. Although our study was strongly limited by the low rate of schoolchildren participation, some consideration can be drawn.

Overall, we found a carriage prevalence of 65.8% and seven year-old schoolchildren showed the highest rate of pneumococcal colonization.

In general, both values and trends documented here are in agreement with other studies conducted in Italy [29,30,31,32], although a comparison is difficult due to different population settings and pneumococcal detection methods (i.e., cultural or molecular-based).

Some community- and household-level factors previously shown to have independent effects on childhood pneumococcal carriage [33] were analysed in this project.

Pneumococcal carriage was inversely related to the number of rooms in the house but directly related to household size, thus suggesting that such factors might play an important role in sustaining the overall circulation of pneumococcal strains at the family-level. These findings were in accordance with Menezes et al. [34], who recently described the same pathway among children in an urban community characterized by crowded environments in the households.

The effect of exposure to tobacco smoke on pneumococcal colonization has been debated in the literature and there is an overall agreement that both active and passive smoking are independent risk factors for carriage [35], as well as for invasive *S. pneumoniae* infections [36,37].

In this context, Le Rouzic and colleagues [38] recently reported that cigarette smoke might alter the ability to activate the antigen specific T-cell response of the human immune system against *Streptococcus pneumoniae*.

However, results from different studies substantially reflect the population setting considered. Indeed, Camilli and colleagues [28] indicated a strong association between pneumococcal carriage in children and a smoking mother in the household, while van Hoek et al. [23] recognized both a slight positive effect of passive smoke and consistently higher odds among active smokers aged 18 years or above. In our paper, we found a positive effect of active smoking on pneumococcal carriage, although this was not significant, and the odds progressively increased with the number of cigarettes smoked per day.

Furthermore, other individual and clinical factors such as the lack of physical activities as well as pre-existing respiratory diseases may also increase the risk of being colonized by pneumococcus in our setting. Zuccotti et al. [32], in Italy, found that the occurrence of respiratory tract infections in the previous three months since bacterial detection, might represent a predictor of nasopharyngeal pneumococcal carriage in healthy children aged 3–59 months.

The potential role of pneumococcal vaccination on carriage remains a critical issue and it is still widely discussed in the scientific literature. It may typically depend on the carriage prevalence in the general population, the immunological status of vaccinated subjects and, not least, the time elapsed since the last PCV dose assumption. In this regard, it has been suggested that the rate of colonization consistently declines in the first year from PCV and then gradually increases in the following years [29,39]. Moreover, Principi and colleagues [31] reported a higher proportion of carriers among vaccinated healthy children and adolescents, irrespective of pneumococcal serotypes included either in 7-valent or 13-valent formulations.

Conversely, Zuccotti and colleagues [32] in a larger study found comparable pneumococcal carriage rates in vaccinated and unvaccinated children less than five years of age, as also observed by Lakshman et al. [40] in UK among children aged two to five years living in a largely unvaccinated population, thus suggesting that PCV immunization does not necessarily interact with the microbial persistence in the upper respiratory tract.

The cohort of schoolchildren enrolled in our study reflects the overall high vaccination coverage against pneumococcus achieved in Sicily and, according to their birth cohorts, the participants to the study were mainly immunized with the 7-valent vaccine formulation.

Nevertheless, five of the seven most commonly identified serotypes in this study represented PCV13 serotypes and were detected at similar frequencies from the oropharynx of schoolchildren and their household contacts, irrespective of vaccination status.

These findings are in accordance to those proposed by Principi et al. [31] in Italy, suggesting that no relevant serotype replacement may have occurred in our geographic area, although the cross-sectional design of our study cannot permit to draw conclusions on this topic.

Notwithstanding, this issue is still far from being cleared in light of other studies on carriage performed after PCV7 implementation in childhood, which assessed a lower carriage rate in vaccinated than in unvaccinated subjects together with a consistent reduction of PCV7 serotypes [41,42,43,44].

The multiple carriage of *Streptococcus pneumoniae* has been already reported by several authors [27,45,46], proving that the number of concurrent serotypes detected strongly depends on the technique used [23,47,48], and molecular-based studies, like this study, inevitably provide a higher number of serotypes simultaneously carried in the oropharynx.

In our cohort, almost all subjects had more than one serotype in their oropharynx and, unexpectedly, the total number per individual followed a nearly normal distribution, with three to five concurrent serotypes as the most frequent setting. A similar trend was observed in a rural setting of African children [49], while conflicting dynamics were recently reported in unvaccinated [45] or highly vaccinated population groups [29], among which the number of simultaneous serotypes was higher, and the relative abundance of subjects involved was lower.

Such evidence may have serious implications in increased opportunities for intra-host horizontal gene transfer, which may lead to capsule switch, antibiotic resistance and vaccine escape [45,50].

The most outstanding ecological feature of *S. pneumoniae* in this study was the clustering propensity of serotypes into families. To date, some authors have explored the dynamics of bacterial colonization within only mother-infant pairs, with the mothers as possible source of carriage [49,51]. Conversely, as far as we know, the pattern of spread of *S. pneumoniae* within family clusters was previously investigated in the second half of nineties [52,53,54,55], whereas it was scarcely explored in the PCV era.

Our experiment underlined a consistent proportion of family groups whose members shared the same pattern of pneumococcal serotypes carried by the schoolchildren, suggesting a potential horizontal transfer of bacteria among household contacts also facilitated by crowded settings.

This circumstance may have important implications in the intrafamilial spread of PCV and non-PCV serotypes driven by vaccinated children toward unvaccinated adults and elderly, who may become colonized and, therefore, susceptible to potentially developing pneumococcal disease.

Of course, our study has some limitations. First, it was a pilot study conducted in a healthy population of two selected primary schools and this limited the statistical power of the study. Second, due to the cross-sectional design of this study and because of the recent introduction of PCV13 in Sicily, it may only represent a snapshot of the current situation in a geographic area characterized by high pneumococcal vaccine coverage in childhood and it is unable to predict trend changes in pneumococcal carriage serotypes. Third, it should be mentioned that rt-PCR molecular methods, in some cases, may cross-react with non-PCV serotypes and this could affect the results. However, we are reasonably confident that the reported PCV13 serotypes distribution adequately reflected the local epidemiological scenario, in accordance with the current scientific evidence from other recent Italian studies [28,32].

Nevertheless, the strength of our study is the lack of previous similar experiments, providing us the opportunity to describe the potential role played by the family environment in sustaining both the circulation of pneumococcal serotypes and the horizontal transmission of carriage toward unvaccinated susceptible household contacts.

In conclusion, although the present work aimed to demonstrate the feasibility in our area, these findings provide baseline information on the dynamics of carriage that would be worth considering on a larger scale and further investigated on different target populations.

Further epidemiological studies and the continuous surveillance of targeted populations are needed to improve the understanding of the dynamics of transmission and carriage, in order to implement new control strategies and to improve actual preventive measures.

## 4. Materials and Methods

### 4.1. Study Population

This pilot surveillance study was named BINOCOLO, an Italian acronym for “children and grandparents joined together against pneumococcus”, and it took place between April and October 2014.

Two primary schools located in the metropolitan area of Palermo were selected to participate in the study. In the Italian Education System, primary school consists of three different grade attended by children 6 years old (first grade), 7 years old (second grade), and 8 years old (third grade), respectively.

Following the school manager approval, six classes were randomly identified in one school (two first grades, two second grades and two third grades, respectively), while the other school contributed with four classes of students (two first grades and two third grades, respectively).

The “school-based” recruitment was followed by a “family-based” recruitment of household contacts of the students that joined the project (i.e., brothers and sisters, parents, grandparents, uncles). In this phase, the family clusters of the study were defined and a recapture sampling for missed recruitments at school was carried out. Each cluster included all family members living in the same household.

Two different structured questionnaires were validated and used to collect information among children (7 sections accounting for 80 items) and adults (7 sections accounting for 75 items), including individual and socio-demographic data, lifestyles, underlying medical conditions, influenza-like symptoms, and vaccination history.

Each participant (child/adult) was requested to answer the questionnaire and to be sampled by oropharyngeal swab. According to family cluster willingness, meetings for questionnaire administration and sampling were performed either at the University Hospital of Palermo “P. Giaccone”, Department of Health Promotion Sciences and Mother-Child Care “G. D’Alessandro” at the University of Palermo (Sicily), or at their home. Each participant received a testimonial gadget at the end of the study.

### 4.2. Ethics Statement

Parents and cohabiting relatives of the schoolchildren were convened for an informative meeting, which was held in the presence of both the manager and qualified teachers of participating schools.

During each meeting, objectives, methods, design and expected results of the study were declared and a leaflet that synthetically reproduced the content of the presentation and working group contacts was also distributed. At the end of each meeting, parents and all members of participating families were asked to give formal written consent for themselves and for their children.

The study was approved by the medical ethics committee of the University Hospital of Palermo with the protocol number 13 (11 December 2013).

### 4.3. Laboratory Methods

Oropharyngeal samples were collected from each participating subject. After collection, the swabs were placed in skim-milk-tryptone-glucose-glycerin (STGG) transport medium according to WHO guidelines [56,57] and stored at −80 °C until further analysis.

Bacterial DNA was extracted and purified using the QIAamp DNA mini kit (Qiagen, Valencia, CA, USA) following the manufacturer’s protocol with a few modifications. Briefly, 180 μL of each sample was added with 80 μL of lysozyme (20 mg/mL in 20 mM Tris-HCl, pH 8.0, 2 mM EDTA, 1.2% Triton) and incubated at 37 °C for 30 min. Following the addition of 20 μL of proteinase K (0.5 mg/mL in Tris-HCl 20 mM, pH 8.0) and 200 μL of the lysis buffer provided with the kit, the mixture was further incubated at 56 °C per 30 min, and the enzyme subsequently denatured at 95 °C for 15 min. All other steps were followed as per the manufacturer’s instructions to a final volume of 100 μL elution buffer.

Carriage of *Streptococcus pneumoniae* was evaluated by means of a single-plex real-time PCR (rt-PCR) assay for the detection of pneumococcal autolysin (*lytA*) gene sequence [57], and a sample was assumed to be negative if there was no increase in fluorescent signal after 40 rt-PCR cycles.

All *lytA* positive samples were included in serotyping analysis by means of single-plex rt-PCR assays for the detection of specific genetic segments of pneumococcal serotypes/serogroups covered by the 13-valent conjugate vaccine (1, 3, 4, 5, 6A/B, 7A/F, 9V/A, 14, 18B/C, 19A, 19B/F, 23F), as previously described by Azzari et al. [47]. To this end, all molecular tests were carried out in duplicate, including both negative and plasmid positive controls of each PCV13 serotype.

### 4.4. Statistical Analysis

Descriptive statistics were used to summarize each of the socio-demographic and clinical variables included in the questionnaires (counts, percentages, median and interquartile range, as appropriate).

The study population was stratified into six age groups. In detail, children were grouped according to the age range for school attendance (5–6, 7 and 8 years old, respectively), while the other subjects were arbitrarily clustered into further three age groups including teenagers/young adults (12–25 years old), adults (26–49 years old) and older adults/elderly (≥50 years old).

Moreover, an indicator of the level of crowding in a private dwelling was identified—the “crowding index”—with the aim of exploring the possible relationships between factors relating to a close proximity of co-habiting individuals (i.e., the number of household members, the number of rooms in the dwelling) and the pneumococcus carriage within each family cluster. To this end, a dummy variable was arbitrarily defined in order to represent a group of families consisting of three persons or more and living in a dwelling with four rooms or less.

Therefore, potential risk factors for pneumococcal carrier status were investigated by univariate logistic regression analysis and the strength of association was expressed as odds ratios (ORs) and 95% confidence intervals (CI).

All of the analyses with *p-*values of 0.05 or less were considered to be statistically significant (two tailed). Data were processed with the STATA MP statistical software package v14.1 for Apple™ (StataCorp, College Station, TX, USA).

## Figures and Tables

**Table 1:** Demographics, clinical characteristics and vaccination status of study participants.

Study Population	Total	School Children	Family Members	
			<12 Years	≥12 Years
**Study participants (*n* (%))**	146	36 (24.7)	18 (12.3)	92 (63.0)
**Age (years; median (IQR))**	31.0 (36.0)	7 (2.0)	5 (8.0)	39.0 (23.0)
Age-group (years)				
5–6	22 (15.1)	12 (33.3)	10 (55.6)	0
7	9 (6.2)	8 (22.2)	1 (5.5)	0
8–11	23 (15.7)	16 (44.5)	7 (38.9)	0
12–25 (teenagers/young adults)	12 (8.2)	0	0	12 (13.1)
26–49 (adults)	52 (35.6)	0	0	52 (56.5)
≥50 (older adults/elderly)	28 (19.2)	0	0	28 (30.4)
**Sex**				
Male	64 (43.8)	23 (63.9)	10 (55.6)	31 (33.7)
Female	82 (56.2)	13 (36.1)	8 (44.4)	61 (63.3)
**Occupation (family members: ≥12 years of age, *n* = 92)**				
None	58 (63.0)			58 (63.0)
Employed	34 (37.0)			34 (37.0)
**Level of education (family members: ≥12 years of age, *n* = 92)**				
None	10 (10.9)			10 (10.9)
Primary education	15 (16.3)			15 (16.3)
Lower secondary education	40 (43.5)			40 (43.5)
Upper secondary education	23 (25.0)			23 (25.0)
Academic degree	4 (4.3)			4 (4.3)
**Smoking habit (family members: ≥12 years of age, *n* = 92)**				
Non-smokers	48 (52.2)			48 (52.2)
Passive smokers (*n* = 48)	16 (33.3)			16 (33.3)
Active smokers	44 (47.8)			44 (47.8)
Age of starting smoking (year; median (IQR))	15 (4.5)			15 (4.5)
Number of cigarettes/cigars (*n*/day; median (IQR))	15 (45)			15 (45)
**Physical activity**				
Hours/week (*n*; median (IQR))	3 (3)	2 (1)	2 (1)	5 (6)
Yes	23 (15.7)	8 (22.2)	4 (22.2)	11 (12.0)
**Clinical features**				
Pre-existing diseases	78 (53.4)	8 (22.2)	2 (11.1)	68 (73.9)
Diabetes	2 (2.6)	0	0	2 (2.9)
Hypertension	22 (28.2)	0	0	22 (32.4)
Heart diseases	5 (6.4)	0	0	5 (7.3)
Chronic bronchitis/emphysema	8 (10.2)	0	0	8 (11.8)
Other	41 (52.6)	8 (100.0) ^#^	2 (100.0) ^#^	31 (45.6)
**Vaccination status**				
Pneumococcal	46 (31.5)	31 (86.1) ^∆^	13 (72.2) ^∆∆^	2 (2.2) ^∆∆∆^
Complete vaccination schedule (*n* = 46)	41 (89.1)	30 (96.8)	10 (76.9)	1 (50.0)
Hexavalent	55 (38.7)	34 (94.4)	18 (100.0)	3 (3.3)
MMR	54 (37.0)	33 (91.7)	18 (100.0)	3 (3.3)
Varicella	47 (32.2)	29 (80.6)	15 (83.3)	3 (3.3)
Meningococcal C	13 (8.9)	5 (13.9)	7 (38.9)	1 (1.1)
Influenza (last 12 months)	8 (5.5)	1 (2.8)	0	7 (7.6)

IQR: interquartile range; Hexavalent: diphtheria, tetanus, acellular pertussis, *Haemophilus influenzae* type B, poliovirus, and hepatitis B; MMR: measles, mumps, and rubella; ^#^ 50%, with allergy-related diseases; ^∆^ 7-valent pneumococcal conjugate vaccine (PCV7): 24 subjects; PCV7 + PCV13: 7 subjects; ^∆∆^ PCV7: 5 subjects; PCV7 + PCV13: 3 subjects; PCV13: 5 subjects; ^∆∆∆^ PCV7: 1 subjects; PPV23: 1 subjects.

**Table 2:** Main socio-demographic characteristics and vaccination status of the study population, according to pneumococcal colonization (*lytA*-pos vs. *lytA*-neg).

Study Population	Pneumococcal Colonization (*lytA*-pos)		*p*-Value	Odds Ratio (95% CI)
	No	Yes		
Study participants (*n* = 146), (*n* (%))	50 (34.2)	96 (65.8)		
Schoolchildren	8 (22.2)	28 (77.8)	0.084	2.161 (0.901–5.185)
Family members	42 (38.2)	68 (61.8)		(reference)
**Age-group (years)**				
5–6	8 (36.4)	14 (63.6)	0.833	1.132 (0.357–3.587)
7	1 (11.1)	8 (88.9)	0.145	5.176 (0.566–47.323)
8–11	5 (21.7)	18 (78.3)	0.184	2.329 (0.669–8.112)
12–25	2 (16.7)	10 (83.3)	0.175	3.235 (0.593–17.658)
26–49	23 (44.2)	29 (55.8)	0.670	0.816 (0.320–2.079)
≥50	11 (39.3)	17 (60.7)		(reference)
**Sex**				
Male	23 (35.9)	41 (64.1)		(reference)
Female	27 (32.9)	55 (67.1)	0.704	1.143 (0.574–2.273)
**Crowding index**				
Low	37 (40.2)	55 (59.8)		(reference)
High	13 (24.1)	41 (75.9)	0.049	2.121 (1.002–4.492)
**Smoking habit (family members: ≥12 years of age, *n* = 92)**				
Non-smokers	23 (46.0)	27 (54.0)		(reference)
Active smokers	13 (30.9)	29 (69.1)	0.143	1.900 (0.805–4.484)
Number of cigarettes/cigars per day				
<5 cigarettes/day	22 (39.3)	34 (60.7)		(reference)
≥5 cigarettes/day	14 (38.9)	22 (61.1)	0.970	1.017 (0.431–2.399)
<10 cigarettes/day	28 (44.4)	35 (55.6)		(reference)
≥10 cigarettes/day	8 (27.6)	21 (72.4)	0.127	2.100 (0.809–5.451)
<15 cigarettes/day	32 (43.8)	41 (56.2)		(reference)
≥15 cigarettes/day	4 (21.0)	15 (79.0)	0.078	2.927 (0.885–9.678)
<20 cigarettes/day	33 (43.4)	43 (56.6)		(reference)
≥20 cigarettes/day	3 (18.8)	13 (81.2)	0.078	3.326 (0.875–12.634)
**Physical activity**				
Yes	9 (39.1)	14 (60.9)		(reference)
No	41 (33.3)	82 (66.7)	0.591	1.286 (0.514–3.218)
**Pre-existing respiratory diseases**				
No	30 (38.5)	48 (61.5)		(reference)
Yes	20 (29.4)	48 (70.6)	0.251	1.500 (0.750–2.999)

Crowding index—“High” identifies families consisting of three persons or more and living in a dwelling with four rooms or less; Respiratory co-morbidities—At least one of the following disease: asthma, chronic bronchitis, nasal obstruction, allergic rhinitis, sinusitis, otitis, tonsillectomy, adenoidectomy.

**Table 3:** Intrafamilial sharing of pneumococcal serotypes, expressed as percentage of detected serotypes in common between household contacts and student, selected as reference.

ID Family Number	Student Age (Reference Case)	Household Size	Intrafamilial Sharing (%)
14	8	2	100.0
23	9	5	90.9
2	8	5	90.5
11	6	4	84.6
34	7	7	81.2
12	8	3	75.0
25	6	3	75.0
33	9	2	75.0
16	7	7	71.4
3	9	5	70.8
20	8	3	69.2
6	8	4	68.7
28	11	4	68.7
19	8	9	65.2
26	7	3	60.0
27	6	5	50.0
10	7	5	47.0
30	8	4	46.7
5	7	3	44.4
21	6	5	42.9
17	8	4	25.0
31	7	4	18.2
1	8	5	0
4	6	2	0
7	6	4	0
8	6	3	0
9	7	3	0
13	7	4	0
15	6	6	0
18	6	3	0
22	8	1	0
24	8	5	0
29	6	3	0
32	8	5	0
35	6	2	0
36	6	4	0

**Table 4:** Socio-demographic variables and pneumococcal intrafamilial sharing, according to low (≤50%) and high score (>50%).

	Intrafamilial Sharing		*p*-Value	Odds Ratio (95% CI)
	Low	High		
**Family clusters (*n* = 36), (*n* (%))**	**21 (58.3)**	**15 (41.7)**		
**Crowding index**				
Low	15 (62.5)	9 (37.5)		(reference)
High	6 (50.0)	6 (50.0)	0.475	1.667 (0.410–6.767)
**Household size**				
≤2 persons	3 (60.0)	2 (40.0)		(reference)
>2 persons	18 (58.1)	13 (41.9)	0.935	1.083 (0.158–7.435)
≤3 persons	9 (60.0)	6 (40.0)		(reference)
>3 persons	12 (57.1)	9 (42.9)	0.864	1.125 (0.292–4.326)
≤4 persons	15 (65.2)	8 (34.8)		(reference)
>4 persons	6 (46.1)	7 (53.9)	0.269	2.188 (0.546–8.761)
≤5 persons	20 (62.5)	12 (37.5)		(reference)
>5 persons	1 (25.0)	3 (75.0)	0.184	5.000 (0.466–53.682)

Crowding index: “High” identifies families consisting of three persons or more and living in a dwelling with four rooms or less.

## Abbreviations

rt_PCR	real-time PCR
OR	odds ratio
IQR	interquartile range
IPD	invasive pneumococcal disease
PCV7	pneumococcal 7-valent conjugate vaccine
PCV13	pneumococcal 13-valent conjugate vaccine
PPV23	pneumococcal 23-valent polysaccharides vaccine
